# Influence of non-steroidal anti-inflammatory drugs on *Drosophila melanogaster* longevity

**DOI:** 10.18632/oncotarget.5118

**Published:** 2015-08-07

**Authors:** Anton Danilov, Mikhail Shaposhnikov, Oksana Shevchenko, Nadezhda Zemskaya, Alex Zhavoronkov, Alexey Moskalev

**Affiliations:** ^1^ Engelhardt Institute of Molecular Biology, Russian Academy of Sciences, Moscow, Russia; ^2^ Institute of Biology of Komi Science Center of Ural Branch of RAS, Syktyvkar, Russia; ^3^ Syktyvkar State University, Syktyvkar, Russia; ^4^ Center for Pediatric Hematology, Oncology and Immunology, Moscow, Russia; ^5^ Moscow Institute of Physics and Technology, Dolgoprudny, Russia

**Keywords:** longevity, anti-inflammatory drugs, Drosophila, lifespan, geroprotector, geotarget

## Abstract

Most age-related diseases and aging itself are associated with chronic inflammation. Thus pharmacological inhibition of inflammatory processes may be effective antiaging strategy. In this study we demonstrated that treatment of *Drosophila melanogaster* with 10 non-steroidal anti-inflammatory drugs (NSAIDs: CAY10404, aspirin, APHS, SC-560, NS-398, SC-58125, valeroyl salicylate, trans-resveratrol, valdecoxib, licofelone) leads to extension of lifespan, delays age-dependent decline of locomotor activity and increases stress resistance. The effect of the lifespan increase was associated with decrease of fecundity. Depending on the concentration, NSAIDs demonstrated both anti- and pro-oxidant properties in *Drosophila* tissues. However, we failed to identify clear correlation between antioxidant properties of NSAIDs and their pro-longevity effects. The lifespan extending effects of APHS, SC-58125, valeroyl salicylate, trans-resveratrol, valdecoxib, and licofelone were more pronounced in males, valdecoxib and aspirin - in females. We demonstrated that lifespan extension effect of NSAIDs was abolished in flies with defective genes involved in Pkh2-ypk1-lem3-tat2 pathway.

## INTRODUCTION

Chronic inflammation is one of the main processes that causes disruption of normal functioning of tissues and contributes to age-associated diseases and aging [1, 2]. There is a popular industry term «inflammaging», which is used to describe aging associated with the development of chronic inflammation [3]. Inflammation is accompanied by increase of the activity of pro-inflammatory pathways with age [4, 5]. Inflammatory cause of different age diseases: metabolic syndrome, Alzheimer's disease and Huntington's disease [6-8]. Pharmacological and genetic inhibition of inflammatory processes is considered as effective and proven anti-aging strategy [9].

Non-steroidal anti-inflammatory drugs (NSAIDs) prevent age-associated features and increases the lifespan in various model organisms including yeast [10], nematodes [11], flies [10], and mice [12, 13]. NSAIDs are also effective in treatment of the neurodegenerative disorders like Alzheimer's disease and Huntington's disease [8, 14-16]. NSAIDs demonstrate anticancer effects, tumor suppression and apoptosis stimulation [7, 17, 18]. Most NSAIDs are traditionally considered to be inhibitors of cyclooxygenases (COX), which induce inflammation activity via catalysis of prostanoid biosynthesis [19].

However, according to growing number of recent publications, NSAIDs have multiple molecular targets, and the range of their activity may be broader than COX inhibition [17, 20].

Many studies have experimentally demonstrated that NSAIDs have antioxidant activity via anti-radical activity and membrane-stabilizing action. Anti-radical activity of NSAIDs is mediated by free radical scavenging and antioxidant enzyme activation [21]. NSAIDs exhibit antioxidant activity on model membranes [22], in cells [23, 24], as well as on the organismal level [25, 26].

Anti-inflammatory effects of rapamycin and resveratrol may be associated with pharmacological stimulation of autophagic activity via mTOR inhibition and AMPK activation, respectively. Autophagy prevents the activation of inflammasomes and induction of inflammatory responses [27, 28].

In *Caenorhabditis elegans* NSAID celecoxib was shown to acts directly on 3′-phosphoinositide-dependent kinase-1 (PDK-1), a component of the insulin/IGF-1 signaling cascade to increase lifespan [11]. In yeast *Saccharomyces cerevisiae* the functional homolog of PDK-1 is Pkh1 which is involved in Pkh2-ypk1-lem3-tat2 signaling pathway. Recent studies demonstrated that another NSAID ibuprofen increases lifespan in yeast, nematodes and fruit flies [10]. This effect is dependent on the ability of ibuprofen to inhibit the tryptophan permease Tat2p which is the component of Pkh2-ypk1-lem3-tat2 signaling pathway also [10, 29]. Functional homologs of Pkh2-ypk1-lem3-tat2 signaling pathway are also known in *Drosophila*, including Pkh2/Pdk1 [30], ypk1/S6k [31], lem3/CG8679 [32], and tat2/CG14741 [33], that allows to study the mechanisms of NSAID effects on *Drosophila* model.

The purpose of this study is to explore the geroprotective properties of NSAIDs, their impact on lifespan, life quality (locomotor activity), fecundity, and stress resistance in *Drosophila* model, reveal toxicity, the antioxidant activity and membrane-protective activity of these compounds using *in vitro* and *in vivo* models. In addition it is planned to clarify the role of Pkh2-ypk1-lem3-tat2 signaling pathway in the formation of geroprotective effects.

## RESULTS

### Effects of NSAIDs on longevity

**Figure 1:**
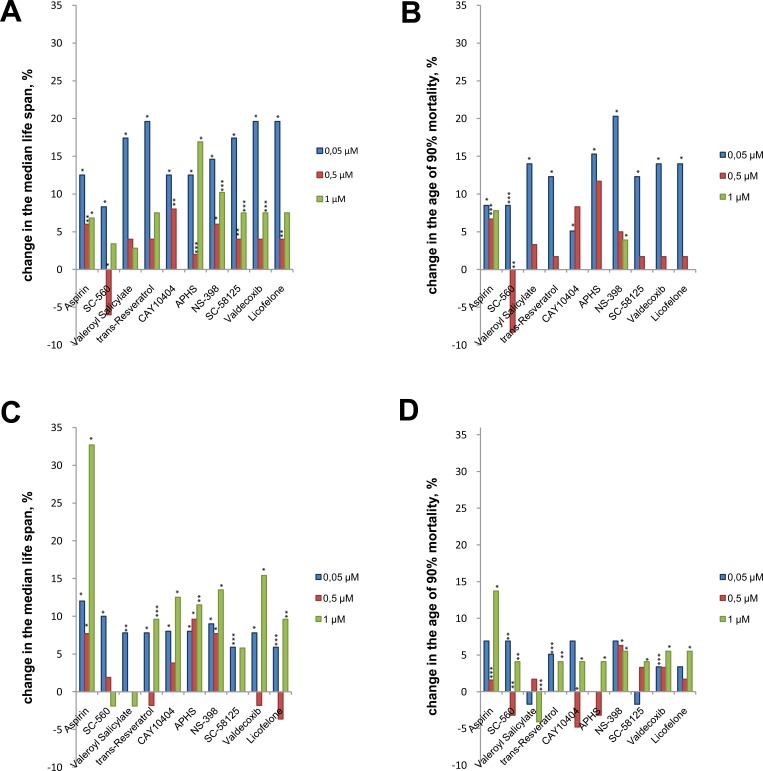
The influence of NSAIDs on the median lifespan and on the age of 90% mortality of *Drosophila melanogaster* Data are represented as percentage changes in comparison with control group. **A.** Males median lifespan. **B.** Males age of 90% mortality. **C.** Females median lifespan. **D.** Females age of 90% mortality. *p < 0.001, **p < 0.01, ***p < 0.05, Gehan-Breslow-Wilcoxon and Mantel-Cox tests for median lifespan; Wang-Allison test for age of 90% mortality.

While exposing the flies to test substances, we observed a significant increase in median survival age and 90% mortality in males and females (Figure 1).

All substances in a concentration of 0.05 μM significant increased the median lifespan of the male by 8.3-19.6% and the age of 90% mortality by 5.1-20.3%. Geroprotective activity of the test NSAIDs in males decreased at concentration 0.5 and 1 μM. At the concentration 0.5 μM, aspirin, CAY10404, APHS, NS-398, SC-58125 and licofelone extended median lifespan by 4-8%. Also, aspirin increased the age of 90% mortality by 6.7%. When exposed to the highest concentration of 1 μM, aspirin, APHS, NS-398, SC-58125 extended median lifespan by 6.8-16.9%, and NS-398 increase the age of 90% mortality by 3.9% (Figure 1A, 1B).

When exposed to NSAIDs in females at concentration of 0.05 μM we observed increases in median lifespan by 5.9-12%. Also SC-560, trans-resveratrol and valdecoxib at this concentration increased the age of 90% mortality by 3.4-6.9%. Aspirin and NS-398 at concentration 0.5 μM extended median lifespan by 7.7 and 9.3% respectively, and the age of 90% mortality by 1.6 and 6.3% respectively. APHS at concentration 0.5 μM increase median lifespan by 9.6%. The most significant effects on the longevity of females we observed when exposed to NSAIDs at concentration of 1 μM. Aspirin at this concentration extended median lifespan by 32.7%. Trans-resveratrol, CAY10404, APHS, NS-398, valdecoxib and licofelone at concentration of 1 μM increased median lifespan by 9.6-15.4%. All test substances except valeryl salicylate increased the age of 90% mortality by 4.1-13.7% (Figure 1C, 1D).

Some of the substances we tested reduced lifespan of flies. SC-560 at a concentration of 0.5 μM significantly reduced the median lifespan of males by 6% and decreased the age of 90% mortality in males by 8.3%, and in females by 3.2% Valeryl salicylate at a concentration of 1 μM reduced the age of 90% mortality of females by 4.1%. CAY10404 at a concentration of 0.5 μM reduced the age of 90% mortality of female by 4.8% (Figure 1A, 1B, 1C, 1D).

### Effects of NSAIDs on fly feeding

According to the feeding assay flies ate less paste containing SC-560 (37.3% lower level of fluorescence), NS-398 (43.1% lower), SC-58125 (44% lower). On the contrary, we observed a higher level of fluorescence in the flies, when aspirin (58.6%) was added into the paste. In other experiment variants we observed no change in the amount of food consumed. Regression analysis did not reveal statistically significant correlation between the amount of food consumed and the changes in lifespan (Supplemental Figure 1).

### Effects NSAIDs on locomotor activity and fertility

Analysis of locomotor activity showed an increase activity in the test for negative geotaxis of males in the last measuring point on the 45th day. Females showed no significant changes in the negative geotaxis test when exposed to NSAIDs.

**Figure 2:**
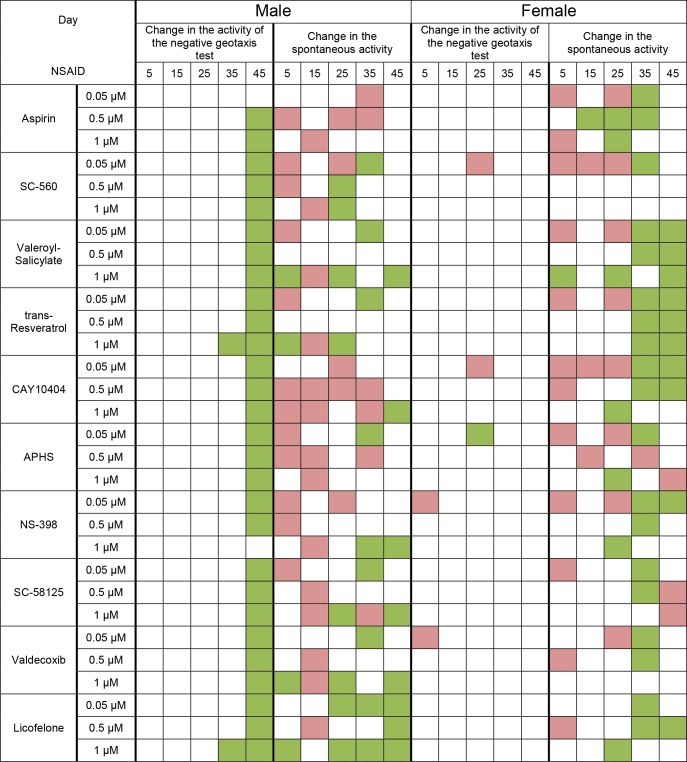
Effect of NSAIDs on the spontaneos activity and the activity in the negative geotaxis test of *Drosophila melanogaster* Green cell - increase of parameter, red cell - decrease of parameter (*p* < 0.05 according to chi-square test), white cell - no significant change in parameter revealed.

We often observed a decrease in spontaneous activity of males and females in the first half of life in 5-25 days. However, in 35 and 45 days, spontaneous activity, in most embodiments a exposure was equal to control or higher than control values. Likofelon had the strongest effect on the conservation and improvement of locomotor function with age (Figure 2).

**Figure 3:**
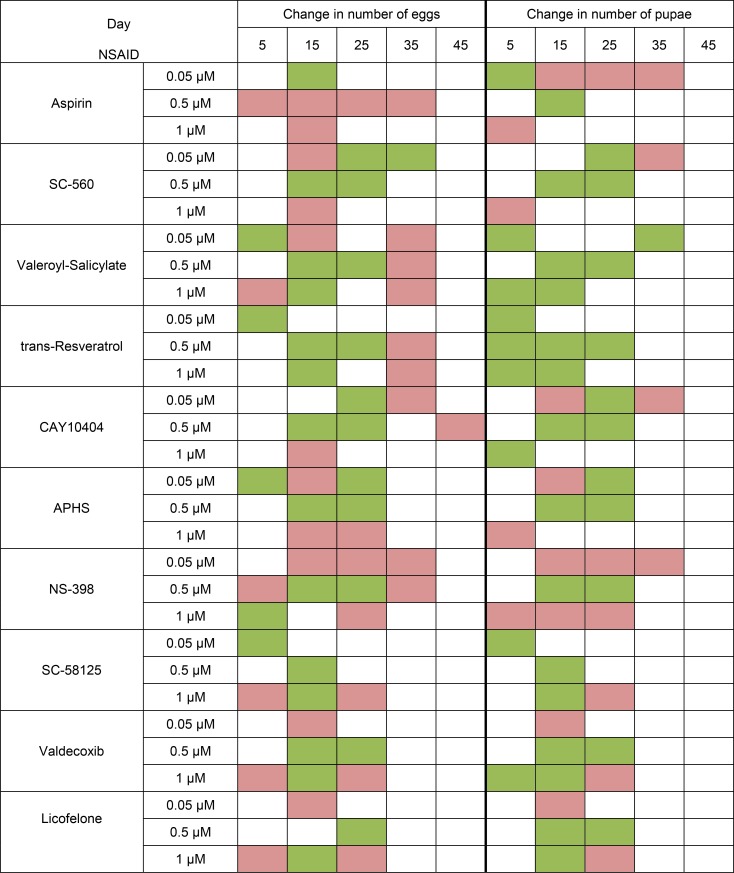
Effect of NSAIDs on fecundityof *Drosophila melanogaster* females Green cell - increase of parameter, red cell - decrease of parameter (*p* < 0.05 according to chi-square test), white cell - no significant change in parameter revealed.

Test substances have different effects on female fertility. We observed an increase in fertility in the middle of the measurement time when exposed to almost all substances. However, we did not find the extension of the reproductive period females. It may be noted that NSAIDs at concentration of 0.05 μM and especially the 0.5 μM were more positive effects on female fertility. In contrast, NSAIDs at concentration of 1 μM had more negative effects on fertility. Some substances with most geroprotective properties often had a fairly strong negative effect on fertility. It was aspirin, APHS and NS-398 (Figure 3).

### Effects of NSAIDs on stress resistance and level of lipid peroxidation products

Preliminary experiments have shown that all tested compounds at a concentration of 0.5 μМ were not toxic to red blood cells, the hemolysis level in the presence of the compounds relative to spontaneous hemolysis was 1.17.

**Figure 4:**
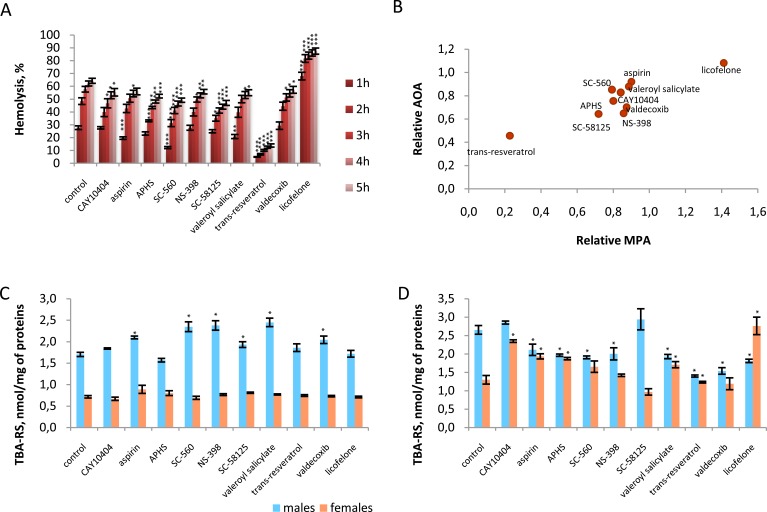
Antioxidant activity of tested compounds in different model systems **A.** NSAIDs (0.5 μМ) effects on the erythrocytes oxidative hemolysis (5 h incubation); **B.** Correlation between the relative membrane-protecting activity (MPA) and the relative antioxidant activity (AOA) of NSAIDs in erythrocytes oxidative hemolysis; **C.** Effect of NSAIDs (0.05 μM) on the content of secondary products of lipid peroxidation (TBA-RS) in tissues homogenates of *Drosophila*; **D.** Effect of NSAIDs (1 μM) on the content of secondary products of lipid peroxidation (TBA-RS) in tissues homogenates of *Drosophila.* *p < 0.05; ***p < 0.001, Mann-Whitney test.

The results of the comparative evaluation of the membrane-protective activity are shown in Figure 4A. Nine of the ten compounds studied were able to protect cells from death under stress of acute oxidative stress. Resveratrol proved to be the most active one in this respect. Only licofelone, a complex inhibitor of cyclooxygenase (COX) and lipoxygenase did not possess the membrane-protective activity; however, it statistically significantly increased the degree of the induced hemolysis. Relative values of the membrane-protective and antioxidant activity have been closely correlated (Rs = 0.782, *p* = 0.008, Figure 4B).

Biologically active compounds can have an impact on organism's lifespan, indirectly, through a complex of different mechanisms. Since a vast majority of the compounds investigated are not only COX inhibitors, they also have a powerful antioxidant activity, it is possible that this property could also affect the results obtained when evaluating the parameters of lifespan of model animals. In order to analyze the contribution of the antioxidant and membrane-protectiveactivity of NSAIDs on lifetime variation in *Drosophila* maintained on a medium supplemented with test compounds at 0.05, 0.5 and 1 μМ concentrations, an appropriate regression analysis was performed. However, none of the cases were statistically significant for an association between the antioxidant/membrane-protective activity of the compounds and the parameters characterizing the *Drosophila* lifespan (median lifespan and age of 90% mortality). Thus, among all compounds studied resveratrol possessed the most significant antioxidant and membrane-protective activity; however, supplementing the nutrient medium with this compound did not lead to a dramatic increase in the lifespan of model animals.

An analysis of the secondary products of lipid peroxidation in *Drosophila* homogenates has demonstrated that at both concentrations (0.05 and 1μM) the test compounds have a more considerable effect on males. At the same time males are characterized by a higher level of lipid peroxidation. Furthermore, the effect of the compounds significantly depended on both the concentration and the sex of animals. Previously, we observed a similar phenomenon in relation to other biologically active substances (BAS) in experiments conducted on laboratory mice [34].

For example, maintenance of flies on the nutrient medium supplemented with NSAIDsat a concentration of 1 μM in most cases resulted in a statistically significant decrease in the LPO level in male organisms, while in females half of the compounds caused an increase in the secondary products of lipid peroxidation (Figure 4C, 4D).

A use of lower concentrations of NSAIDs in the nutrient medium (0.05 μM) caused an increase in the intensity of lipid peroxidation in male tissues and did not significantly affect females (Figure 4C, 4D). Regression analysis showed no association between the intensity of lipid peroxidation in *Drosophila* tissues and the parameters characterizing their lifespan.

Thus, despite the fact that the vast majority of the NSAIDs examined in this study have prominent antioxidant properties, other mechanisms of their pharmacological activity are implemented as well. This underlies an ambiguous effect on such an integral indicator, as lifespan.

### Stress resistance analysis

**Figure 5:**
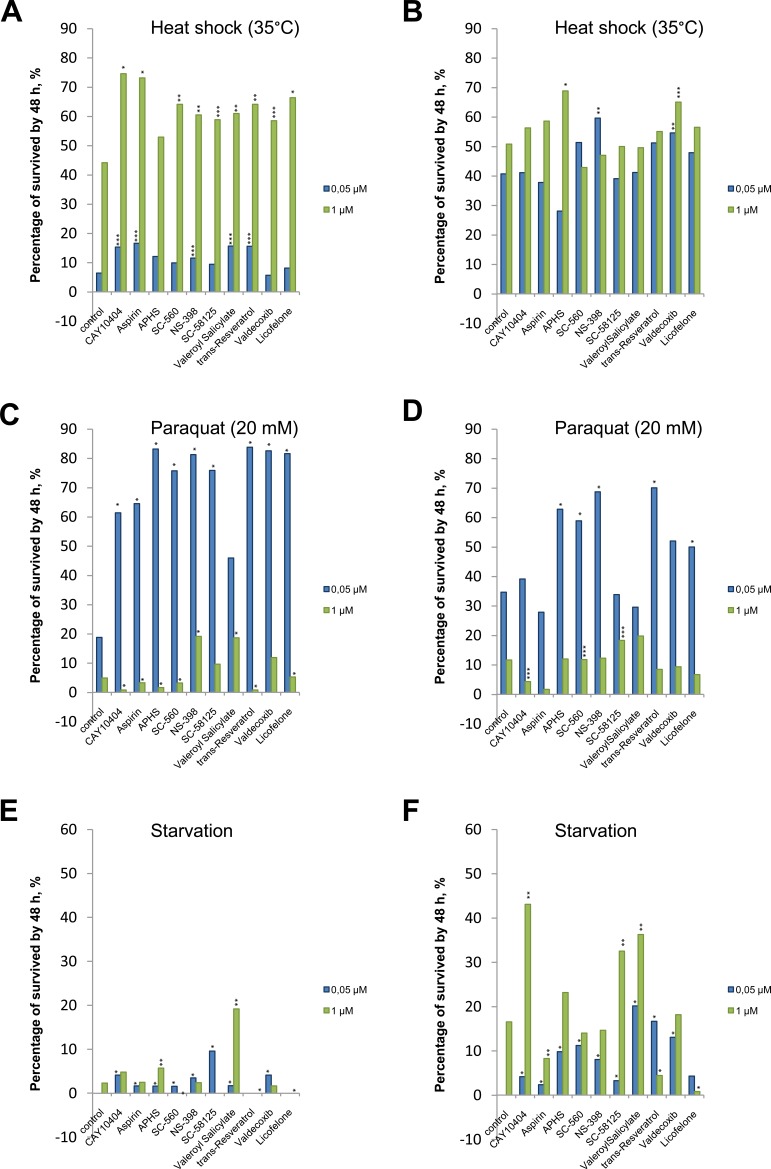
The influence of NSAIDs on the percentage of survived flies after 48 hour of stress impact Data are represented as percentage. **A.** Males, heat shock (35°C). **B.**Females, heat shock (35°C). **C.**Males, oxidative stress (paraquat 20 mM). **D.**Females, oxidative stress (paraquat 20 mM). **E.**Males, starvation. **F.**Females, starvation. **p < 0.001, **p < 0.01, ***p < 0.05, Fisher's exact test.

It was demonstrated that in males NSAIDs at a concentration of 0.05 μM significantly improved survival ratio upon exposure to paraquat, and at a concentration of 1 μM they significantly improved it under heat shock. There was no significant increase in survival during starvation. Upon exposure to CAY10404, aspirin, APHS, SC-560 and trans-resveratrol at concentrations of 1 μM, we observed a decrease in survival ratio under oxidative stress, where as upon exposure to SC-560, SC-58125, trans-resveratrol, licofeloneat a concentration of 1 μM we observed such a decrease during starvation (Figure 5).

Studies of the resistance of females to exogenous stresses demonstrated that NSAIDs at concentrations of 1 and 0.05 μM significantly increased survival ratios upon exposure to paraquat, under heat shock and during starvation. Aspirin at a concentration of 0.05 μM reduced the survival ratio of females under heat shock, at a concentration of 1 μM it reduced the survival ratio in starvation, and at the concentrations of 1 μM and 0.05 μM it reduced the survival ratio under oxidative stress. APHS significantly reduces the survival ratio of females under heat shock at a concentration of 0.05 μM, while SC-560 and NS-398 significantly reduce it at a concentration of 1 μM. Valeryl salicylate reduces the survival ratio of females under oxidative stress at a concentration of 0.05 μM, whereas CAY10404, trans-resveratrol and licofelone reduce it at a concentration of 1 μM. Trans-resveratrol and licofelone at a concentration of 1 μM significantly reduce the resistance of females to starvation (Figure 5).

### The role of Pkh2-ypk1-lem3-tat2 pathway

In order to understand the mechanisms of geroprotective properties of NSAIDs we studied the effects of aspirin, valdecoxib, and NS-398 on the lifespan of *Drosophila* defective in Pkh2-ypk1-lem3-tat2 signaling pathway.

**Figure 6:**
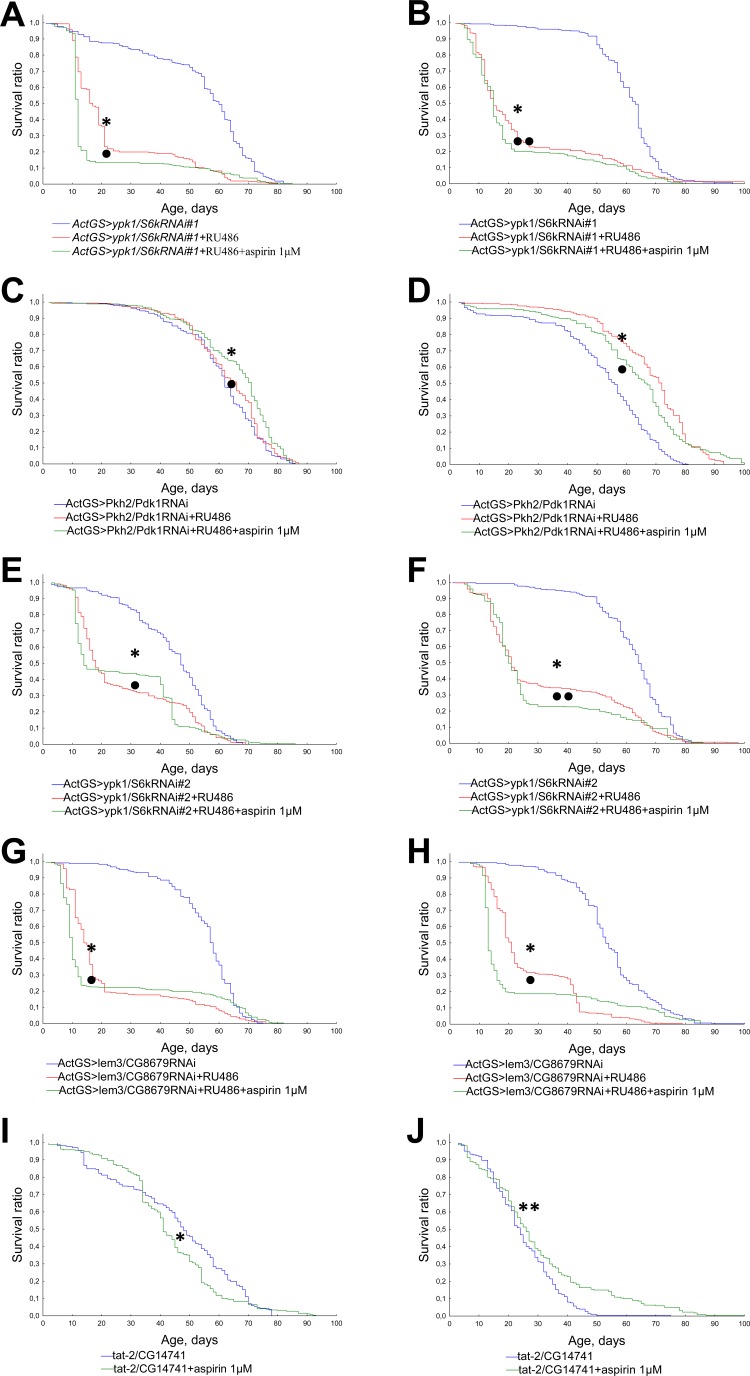
Effect of aspirin (1 μM) on the lifespan of *Drosophila* with down-regulated Pkh2-ypk1-lem3-tat2 signaling pathway **A.***ActGS>ypk1/S6kRNAi#1* males. **B.***ActGS>ypk1/S6kRNAi#1* females. **C.***ActGS>Pkh2/Pdk1RNAi* males. **D.***ActGS>Pkh2/Pdk1RNAi* females. **E.***ActGS>ypk1/S6kRNAi#2* males. **F.***ActGS>ypk1/S6kRNAi#2* females. **G.***ActGS>lem3/CG8679RNAi* males. **H.***ActGS>lem3/CG8679RNAi* females. **I.***tat-2/CG14741* males. **J.***tat-2/CG14741* females. *p < 0.001, **p < 0.05 when comparing flies of intact group without RU486 and NSAIDs treatment with RU486 +NSAID treated group; according to Kolmogorov-Smirnov's test. •*p* < 0.001, ••*p* < 0.05 when comparing RU486 treated group to RU486 +NSAIDs treated group; according to Kolmogorov-Smirnov's test.

**Figure 7:**
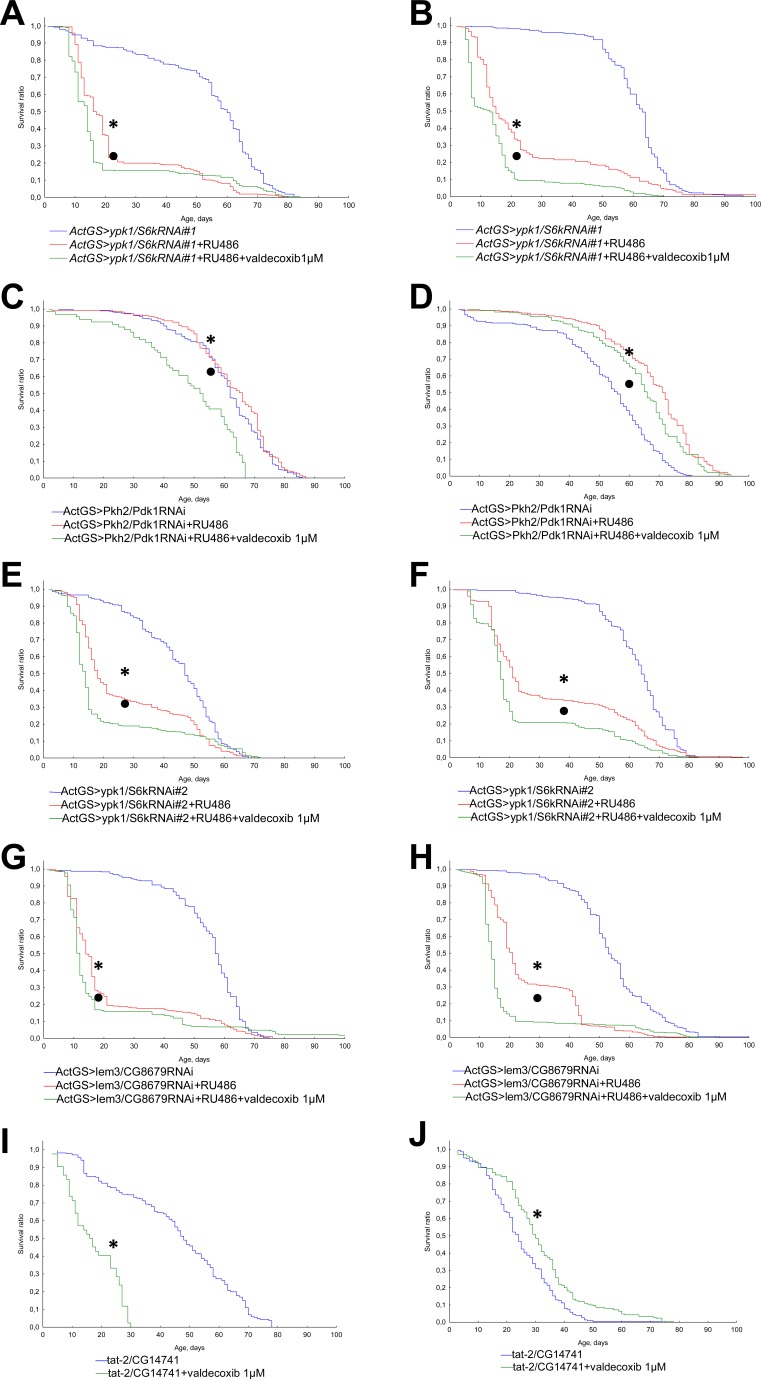
Effect of valdecoxib (1 μM) on the lifespan of *Drosophila* with down-regulated Pkh2-ypk1-lem3-tat2 signaling pathways **A.***ActGS>ypk1/S6kRNAi#1* males. **B.***ActGS>ypk1/S6kRNAi#1* females. **C.***ActGS>Pkh2/Pdk1RNAi* males. **D.***ActGS>Pkh2/Pdk1RNAi* females. **E.***ActGS>ypk1/S6kRNAi#2* males. **F.***ActGS>ypk1/S6kRNAi#2* females. **G.** *ActGS>lem3/CG8679RNAi* males. **H.** *ActGS>lem3/CG8679RNAi* females. **I.** *tat-2/CG14741* males. **J.** *tat-2/CG14741* females. *p < 0.001, **p < 0.05 when comparing flies of intact group without RU486 and NSAIDs treatment with RU486 +NSAID treated group; according to Kolmogorov-Smirnov's test. •*p* < 0.001, ••*p* < 0.05 when comparing RU486 treated group with RU486 +NSAIDs treated group; according to Kolmogorov-Smirnov's test.

**Figure 8:**
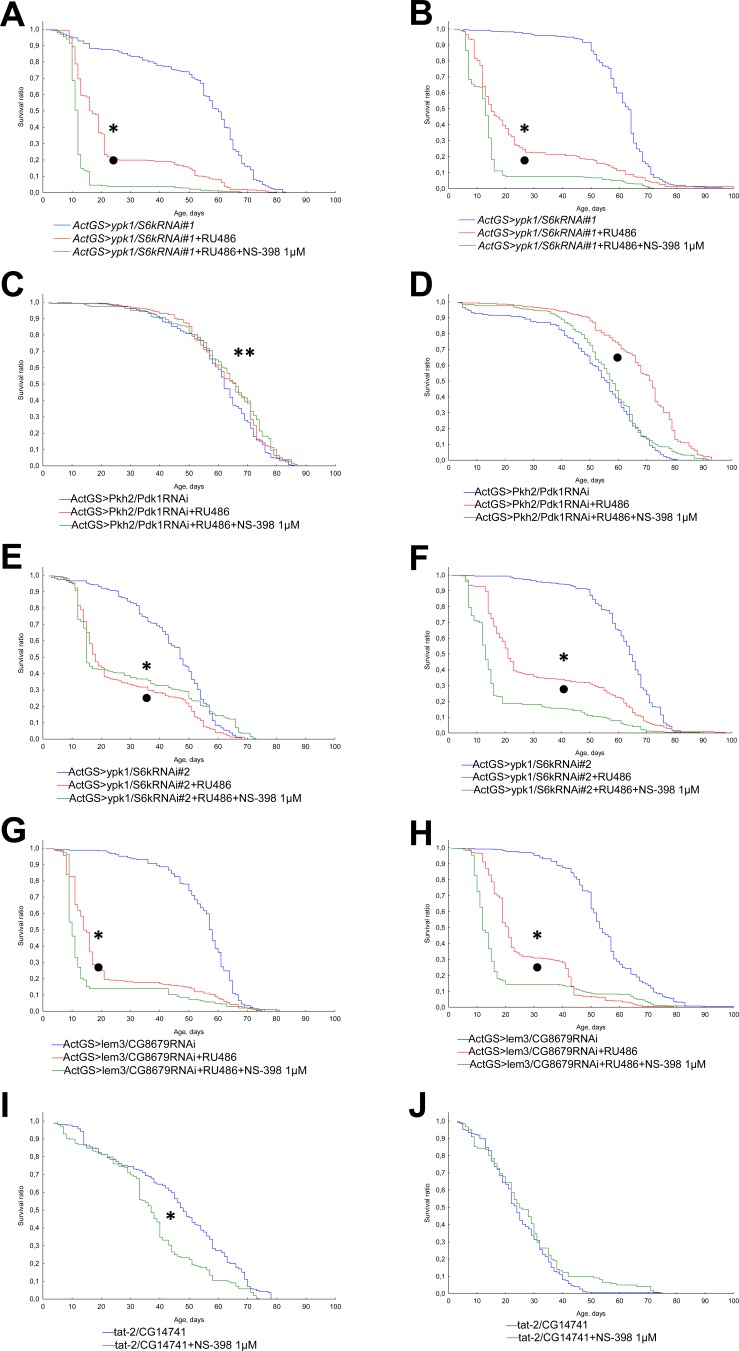
Effect of NS-398 (1μM) on the lifespan of *Drosophila* with down-regulated Pkh2-ypk1-lem3-tat2 signaling pathways **A.***ActGS>ypk1/S6kRNAi#1* males. **B.***ActGS>ypk1/S6kRNAi#1* females. **C.***ActGS>Pkh2/Pdk1RNAi* males. **D.***ActGS>Pkh2/Pdk1RNAi* females. **E.***ActGS>ypk1/S6kRNAi#2* males. **F.***ActGS>ypk1/S6kRNAi#2* females. **G.** *ActGS>lem3/CG8679RNAi* males. **H.** *ActGS>lem3/CG8679RNAi* females. **I.** *tat-2/CG14741* males. **J.** *tat-2/CG14741* females. *p < 0.001, **p < 0.05 when comparing flies of intact group without RU486 and NSAIDs treatment to RU486 +NSAID treated group; according to Kolmogorov-Smirnov's test. •*p* < 0.001, ••*p* < 0.05 when comparing RU486 treated group to RU486 +NSAIDs treated group; according to Kolmogorov-Smirnov's test.

We observed a decrease in lifespan in flies with activated RNA interference of genes *ypk1/S6k*, *Pkh2/Pdk1* and *lem3/CG8679* upon exposure to aspirin, valdecoxib, NS-398 at a concentration of 1 μM (Figures 6, 7, 8). However, in*ActGS>ypk1/S6kRNAi#2* males exposure to NS-398 induced a slight extension in lifespan. In *ActGS>lem3/CG8679RNAi* females we observed an increase in the survival ratio in the second half of life. The treatment with NSAIDs of *tat-2/CG14741* knockout males were characterized by a decreased lifespan, while females, had an extended lifespan.

## DISCUSSION

Thus we investigated the effects of 10 NSAIDs at concentrations of 0.05, 0.5 and 1 μM on *Drosophila* lifespan, life quality (locomotor activity), fecundity, and stress resistance (oxidative stress, heat shock and starvation).

We demonstrated that all studied NSAIDs induced increase in median lifespan (by 4-19% in males and by 2-33% in females), and an age of 90% mortality rate (by 2-20 % in males and by 2-13 % in females). However, treatment of males with 0.5 μM of SC-560 and females with 1 μM of valeroyl salicylate decreased lifespan. The most effective NSAIDs which increased lifespan by more than 15% were APHS (by 17%), SC-58125 (by 17%), valeroyl salicylate (by 17%), trans-resveratrol (by 20%), valdecoxib (by 20%),and licofelone (by 20%) in males, valdecoxib (by15%) and aspirin (by 33%) in females. The most active concentrations were 0.05μM in males and 1 μM in females.

Our data are consistent with the lifespan extending effects of NSAIDs previously obtained in yeast [10, 35], nematodes [10, 11, 36], flies [10], honey bee [37], and in mice [12]. The inhibition of other pro-inflammatory factors such as NF-κB [38] and iNOS [39] increases *Drosophila* lifespan also.

According to the feeding assay analysis flies consumed less food containing some substances (SC-560, NS-398, SC-58125). However, we did not observe any correlation between lifespan and the level of food consumption (Supplemental Figure 1).

In addition, NSAIDs delayed age-dependent decline in locomotor activity. This effect may be associated with neuroprotective action of NSAIDs [14, 15]. As it was shown previously, aspirin reduces age-associated functional declines in *C. elegans* also [36].

We observed a decrease in female fecundity. It may be related to the fact that NSAIDs inhibits the activity of Pxt, a COX-like facilitator of follicle maturationin *Drosophila* [40]. Therefore, the effect of the lifespan increase may be associated with decrease offecundity.

Attention is drawn to the similarity of life-extending effects of various anti-inflammatory drugs (Figure 1). This suggests that non-specific component (hormesis) may also be implicated in triggering these effects [41]. Hormesis is usually associated with the activation of various cellular stress-resistance mechanisms, such as heat shock proteins, antioxidant enzymes, DNA repair mechanisms, immune response, selection of unfit cells and may increase both longevity and stress resistance [42-49].

Therefore, we tested whether NSAIDs might also increase *Drosophila* resistance to oxidative stress, heat shock and starvation. We found that NSAIDs increased resistance to various stresses. Increased resistance to heat shock and starvation may be related to the ability of NSAIDs to inhibit the components of the insulin/IGF-1 signaling [11, 50, 51].

Depending on the concentration and the experimental model NSAIDs demonstrate both anti- and pro-oxidant properties. The antioxidant properties were revealed in the erythrocytes model and *Drosophila* tissues at a concentrations of 0.5 μM and 1 μM, accordingly. However, at a concentration of 0.05 μM we observed pro-oxidant properties of NSAIDs in *Drosophila* males. Thus we failed to find correlation between antioxidant properties of NSAIDs and their antiaging action in *Drosophila*.

Thus, our data support the new paradigm that ROS is not involved in aging under physiological, nonstressed conditions [52]. Numerous experiments on animals also showed that manipulation of antioxidant gene expression often has little effect on life span, and that levels of mitochondrial ROS do not limit it and moreover an increased ROS production correlated with extended life span [53, 54]. Clinical trials demonstrated that current antioxidants do not prolong life and reduction of ROS by antioxidants can even shorten life span [54]. Perhaps this is due to ROS important physiological roles, including regulatory role in cellular signaling pathways, and elimination of ROS may not be favorable for the organism.

Previous studies demonstrated that antioxidant and neuroprotective effects of NSAIDs are associated with inhibition of COX and reduction in the production of reactive oxygen species (ROS) in the metabolism of arachidonic acid, a decrease of the prostaglandin synthesis and a decline of β-amyloid accumulation [8, 14, 15]. Licofelone, due to its conformational similarity with arachidonic acid, is capable of binding to the active sites of both classes of enzymes COX-1 and COX-2, blocking their catalytic activity [55], and normalizing the parameters of lipid peroxidation in brain tissues with an experimental Huntington's syndrome [26].

At the same time, NSAIDs are known to have intrinsic antioxidant activity [22, 24, 56]. For example, a complex biological activity of resveratrol is associated not only with its ability to interact with multiple molecular targets, in particular, cyclooxygenase, but also with a high antioxidant activity due to both a direct elimination of ROS and inhibition of enzymes involved in their formation, as well as enhancement of activity of antioxidant protection enzymes [57]. It has been experimentally demonstrated that resveratrol possesses a high antioxidant activity on model membranes [22], in cells [24, 58, 59], as well as in model organisms [25, 60]. Furthermore, many of the effects of resveratrol are due to an action of its metabolites, and even very low doses of resveratrol can affect an organism through an indirect action [57].

Thus, the antioxidant effect of NSAIDs may be less pronounced on the level of lipid peroxidation due to the absence of COX in *Drosophila*. However intrinsic antioxidant activity of NSAIDs may protect *Drosophila* from acute toxicity of paraquat.

In accordance with the findings available from literature, ibuprofen and celecoxib have targets in yeast (Tat2p) [10], nematodes (Pkh2/PDK-1) [11], involved in Pkh2-ypk1-lem3-tat2 signaling pathway [30]. We therefore studied the role of Pkh2-ypk1-lem3-tat2 signaling pathway in the effects of NSAIDs on lifespan in *Drosophila* model.

In our study, RNA interference of gene *Pkh2/Pdk1* led to an increase in longevity of *Drosophila* females. Our data is consistent with the effects of pharmacological inhibition of Pkh2/PDK-1 in nematode [11]. Simultaneous RNAi of *Pkh2/Pdk 1*and exposure to NSAIDs reduced the positive effect of RNA interference on female longevity. It should be noted that the effects of NSAIDs on flies with other components of Pkh2-ypk1-lem3-tat2 signaling pathway being affected also led to a decrease in longevity. Thus the lifespan extending effects of NSAIDs in *Drosophila* model is mediated by Pkh2-ypk1-lem3-tat2 signaling pathway.

Thus, we demonstrated the ability of 10 NSAIDs (CAY10404, aspirin, APHS, SC-560, NS-398, SC-58125, valeroyl salicylate, trans-resveratrol, valdecoxib, licofelone) to extend lifespan and increase resistance to stress in *Drosophila* accompanied by positive effects on locomotor activity. The lifespan extending effects of APHS, SC-58125, valeroyl salicylate, trans-resveratrol, valdecoxib, and licofelone was more pronounced in males, valdecoxib and aspirin-in females. The effect of the lifespan increase was associated with decrease of fecundity. No correlation was found between the antioxidant properties of NSAIDs and lifespan-extending effect. At the same time, increasing of the survival rate of NSAIDs-treated flies under the influence of paraquat can be associated with the antioxidant activity of substances. The lifespan extension effect of NSAIDs was abolished in flies that are defective in genes of Pkh2-ypk1-lem3-tat2 pathway.

## MATERIALS AND METHODS

### Drosophila melanogaster strains

Strains obtained from the Bloomington *Drosophila* Stock Center were used in this study.

Standard laboratory wild-type strain *Canton-S* (stock #1) was used to test geroprotective properties of NSAIDs.

To investigate the role of *Pkh2-ypk1-lem3-tat2* signaling pathway in NSAIDs effects we used knockout line *tat-2/CG14741*(stock #18847, genotype: *w^1118^; PBac{WH}ATP8B^f05203^*), as well as transgenic RNAi strains: *Pkh2/Pdk1RNAi* (stock #27725, genotype: *y^1^ v^1^; P{TRiP.JF02807}attP2*), *ypk1/S6kRNAi#1* (stocks #41702, genotype: *y^1^ v^1^*; *P{TRiP.HMS02267}attP2*), *ypk1/S6kRNAi#2* (stocks #41895, genotype: y^1^ sc^*^ v^1^; P{TRiP.GL01327}attP2), *lem3/CG8679RNAi* (stock #38348, genotype: *y1 v1; P{TRiP.HMS01816}attP40*). To induce RNAi we used driver line *ActGS* containing the RU486-inducible GAL4 in all cells (stock #9431, genotype: P{hsFLP}12, y^1^ w^*^; *P{UAS-GFP.S65T}Myo31DF^T2^*; *P{Act5C(−FRT)GAL4.Switch.PR}3/TM6B, Tb^1^*).

### Treatment with substances

We greased fly medium by paste of hydrolyzed yeast containing one of the substances. Control untreated animals were fed by yeast past without substances. To make the hydrolyz at yeast were boiled in water bath for 30 minutes. To prepare the 100 ml of paste 50 g of dry yeast per 60 mL of water were used.

Flies were treated throughout their whole lives with the substances as follows: aspirin (2-(acetyloxy)-benzoic acid), valeryl salicylate (2-[(1-oxopentyl)oxy]-benzoicacid), trans-resveratrol ((E)-5-[2-(4-hydroxyphenyl)ethenyl]-1,3-benzenediol), SC-560 (5-(4-chlorophenyl)-1-(4-methoxyphenyl)-3-(3fluoromethyl)-1H-pyrazole), APHS (2-(2-heptynylthio)-phenol-acetate), NS-398 (N-[2-(cyclohexyloxy)-4-nitrophenyl]-methanesolfonamide), SC-58125 (5-(4-fluorophenyl)-1-[4-(methylsulfonyl)-phenyl]-3-(trifluoromethyl)-1H-pirazole), valdecoxib (4-(5-methyl-3-phenyl-4-isoxazolyl)-benzenesulfonamide), CAY10404 (3-(4-methylsulphonylphenyl)-4-phenyl-5-trifluoromethylisoxazole), licofelone (6-(4-chlorophenyl)-2, 3-dihydro-2,2-dimethyl-7-phenyl-1H-pyrrolizine-5-acetic acid) at the concentrations of 0.05, 0.5, 1 μM (Cayman Chemical, USA).

### Feeding assay

To evaluate the effects of NSAIDs on the food intake we used the ﬂuorescein dye (Sigma-Aldrich, USA) as the food tracer. To perform the test, three-to-four day-old females were placed on the paste (with or without NSAIDs in concentration 1 μM) containing 50 μM ﬂuorescein. After 1 h of feeding, the ﬂies were anesthetised and placed in liquid nitrogen for 20 sec. The frozen ﬂies were then brieﬂy vortexed in tube to separate ﬂy heads from the bodies. The ﬂy bodies (5 ﬂies per 1 mL) were then homogenized in distilled water, and the homogenates were centrifuged 2 min at 15,000g. Then 0.8 mL of supernatant was transferred to a new tube, the volume was adjusted to 1.5 mL, and centrifugation was repeated. Before the measurements, the supernatant was diluted with distilled water 1:1. The ﬂuorescence level in resulting solution was then measured on spectroﬂuorimeter Fluorat-02-Panorama (Lumex, Russia) with a ﬂuorescence excitation of 480 nm and an emission registration of 521 nm. Nine independent replicates were performed for each experiment. The level of food intake was estimated on the base of average values of ﬂuorescence intensity. Regression analysis was performed to assess the correlation between the amount of food consumed and the lifespan. To assess the statistical significance of differences fluorescence level was carried out using the t-test.

### Lifespan analysis

To analyze the lifespan, the imagoes of the same age of both sexes were selected. Control and experimental flies were maintained at 25±0.5°C under a 12h light/12h dark cycle and at densities of 30 same sex and age flies per vial containing sugar-yeast medium covered with a yeast paste. Flies were transferred to fresh medium 2 times per week. The number of dead flies was counted daily. The longevity of males and females was analyzed separately. Analysis was carried out in three replications, with 80-120 flies in each.

### Locomotor activity analysis

In order to measure locomotor activity a hardware and software complex «*Drosophila* population monitor» (TriKinetics Inc., USA) was used. Spontaneous locomotor activity was measured (for 3 min), and a negative geotaxis test was performed. The flies were shaken off to the bottom of a tube, and movement was measured for 20 sec in three replications. When evaluating the spontaneous activity, an integral activity of flies for 3 min was registered. For the negative geotaxis test, an arithmetical mean for three replications was calculated to offset an impact of random factors. The measurements in each variant were carried out, as long as there was the sufficient number of living flies to perform the analysis, which was 30 pieces.

### Female fecundity analysis

Fecundity was evaluated by the mean number of eggs and pupae per female. For this purpose, the groups consisting of 10 males and 10 females with the same age were placed into vials with fresh medium. After one day, the flies were transferred to a new medium, and the number of eggs laid was counted. After 10 days pupae formed were counted. A mean fecundity was calculated as a ratio between the number of eggs or pupae and the number of females in a group.

### Stress resistance analysis

To study an effect of heat shock, the flies were placed into an incubator at 35° C.

To study an effect of the oxidative stress, a solution of 20 mM of the pro-oxidant paraquat dissolved in 5% sucrose was used. This oxidative stress medium was applied to a filter paper and placed into vials instead of the nutrient medium.

To study the effect of starvation on the flies they were placed into vials with 2% agar.

### Toxicity, antioxidant activity and membrane-protective activity study

To study the toxicity, antioxidant activity and membrane-protective activity (*in vitro*) of the compounds, an erythrocyte suspension of laboratory mice blood in phosphate buffered saline (PBS, pH 7.4) was used. The toxicity of the compounds was evaluated by their ability to induce the death of erythrocytes (hemolysis). The solution of compounds dissolved in ethanol were added to the erythrocyte suspension at a final concentration of 0.5 μM and incubated at 37° C for 5 h in a thermostatically controlled shaker Biosan ES-20 (Latvia), control samples also contained ethanol. The membrane-protective and antioxidant activities were determined by a degree of inhibition of induced hemolysis and inhibition of accumulation of secondary products of lipid peroxidation in erythrocytes, respectively. For this purpose, after adding of the solutions of the different compounds (final concentration of 0.5 μM) to the erythrocyte suspension the hemolysis was induced by the addition of H_2_O_2_ solution (1.8 mM). Then this reaction mixture was shaken gently while being incubated for 5 h, at 37°C. Every hour an aliquot of the incubation medium was taken and centrifuged for 5 min. The hemolysis degree was determined based on the hemoglobin content in the supernatant using a spectrophotometer Genesys 20 (Thermo Scientific, USA) at λ 524 nm, the percentage of hemolysis was calculated in relation to the total hemolysis of a sample [61-63]. Each experiment was performed in five parallels and two replications.

### Analysis of the level of secondary products of lipid peroxidation (LPO)

The content of the secondary products of lipid peroxidation from a reaction with 2-thiobarbituric acid (TBA-RS) was determined using the spectrophotometer at λ = 532 nm [64] in a modification [65, 66]. To prevent the oxidation of lipids in the process of analysis a butylated hydroxyltoluene (BHT) solution in ethanol (0.01%) was added to the samples. Samples without addition of the TBA solution were used as an external control in order to avoid artifacts of measuring LPO due to the Drosophila eye color. A concentration of TBA-RS products was calculated taking into account the extinction coefficient (1.56 × 10^5^ M^−1^ cm^−1^) and expressed in nmol/mg protein or nmol/mg of fly mass.

### Statistical analysis

Lifespan analyses were carried out using non-parametric methods were utilized. Survival was assessed using Kaplan-Meier survival curves [67]. A median lifespan and age of 90% mortality were calculated. When comparing the survival functions, a modified Kolmogorov-Smirnov's test was applied [68]. To assess the significance of differences for the median lifespan a Gehan-Breslow-Wilcoxon test [69] and Mantel-Cox test [70] were used. To assess a statistical significance of differences in the age of 90% mortality a Wang-Allison test was used [71]. The Kaplan-Meier curves were plotted using a program Statistica, version 6.1 (StatSoft, Inc., USA), the calculation of the lifespan parameters and their statistical analysis were performed in a statistical programming environment R [72].

To assess the statistical significance of differences in resistance to stress-factors, the Fisher's exact test was used [73, 74]. Statistical analyses of the data were performed using OASIS: Online Application for the Survival Analysis of Lifespan Assays [74].

Statistical significance of differences in the fecundity and locomotor activity was evaluated using the χ2 criterion [75].

To process the locomotor activity and fecundity data Microsoft Excel 2010 (Microsoft, USA) was used.

Statistical significance of differences in toxicity, antioxidant and membrane-protecting activity of NSAIDs (*in vitro* erythrocytes model) and content of LPO secondary products in flies was assessed by a non-parametric Mann-Whitney test [76]. Significance was set at *p* < 0.05. Regression analyses were also performed to compare the changes of various parameters tested for using model objects. Spearman's rank correlation coefficient (Rs) was calculated. Analyses were performed by applying software packages Microsoft Office Excel 2007 and Statistica 6.0.

## SUPPLEMENTARY FIGURE



## References

[1] Franceschi C, Campisi J (2014). Chronic inflammation (inflammaging) and its potential contribution to age-associated diseases. The Journals of Gerontology Series A: Biological Sciences and Medical Sciences.

[2] Howcroft TK, Campisi J, Louis GB, Smith MT, Wise B, Wyss-Coray T, Augustine AD, McElhaney JE, Kohanski R, Sierra F (2013). The role of inflammation in age-related disease. Aging (Albany NY).

[3] Stepanova M, Rodriguez E, Birerdinc A, Baranova A (2015). Age-independent rise of inflammatory scores may contribute to accelerated aging in multi-morbidity. Oncotarget.

[4] de Magalhaes JP, Curado J, Church GM (2009). Meta-analysis of age-related gene expression profiles identifies common signatures of aging. Bioinformatics.

[5] Kriete A, Mayo K L, Yalamanchili N, Beggs W, Bender P, Kari C, Rodeck U (2008). Cell autonomous expression of inflammatory genes in biologically aged fibroblasts associated with elevated NF-kappaB activity. Immun Ageing.

[6] Cai D, Liu T (2012). Inflammatory cause of metabolic syndrome via brain stress and NF-kappaB. Aging (Albany NY).

[7] Cusimano A, Azzolina A, Iovanna JL, Bachvarov D, McCubrey JA, D'Alessandro N, Montalto G, Cervello M (2010). Novel combination of celecoxib and proteasome inhibitor MG132 provides synergistic antiproliferative and proapoptotic effects in human liver tumor cells. Cell Cycle.

[8] Breder CD (1997). Cyclooxygenase systems in the mammalian brain. Ann N Y Acad Sci.

[9] López-Otín C, Blasco MA, Partridge L, Serrano M, Kroemer G (2013). The Hallmarks of Aging. Cell.

[10] He C (2014). Enhanced Longevity by Ibuprofen, Conserved in Multiple Species, Occurs in Yeast through Inhibition of Tryptophan Import. PLoS Genet.

[11] Ching TT, Chiang WC, Chen CS, Hsu AL (2011). Celecoxib extends C. elegans lifespan via inhibition of insulin-like signaling but not cyclooxygenase-2 activity. Aging Cell.

[12] Strong R (2008). Nordihydroguaiaretic acid and aspirin increase lifespan of genetically heterogeneous male mice. Aging Cell.

[13] Lee ME, Kim SR, Lee S, Jung YJ, Choi SS, Kim WJ, Han JA (2012). Cyclooxygenase-2 inhibitors modulate skin aging in a catalytic activity-independent manner. Exp Mol Med.

[14] Asanuma M, Miyazaki I, Diaz-Corrales FJ, Ogawa N (2004). Quinone formation as dopaminergic neuron-specific oxidative stress in the pathogenesis of sporadic Parkinson's disease and neurotoxin-induced parkinsonism. Acta Med Okayama.

[15] Black PH (2002). Stress and the inflammatory response: a review of neurogenic inflammation. Brain Behav Immun.

[16] Choi SH, Ai dS, Caracciolo L, Minami SS, Niikura T, Matsuoka Y, Turner RS, Mattson MP, Bosetti F (2013). Cyclooxygenase-1 inhibition reduces amyloid pathology and improves memory deficits in a mouse model of Alzheimer's disease. J Neurochem.

[17] Poole JC, Thain A, Perkins ND, Roninson IB (2004). Induction of transcription by p21Waf1/Cip1/Sdi1: role of NFkappaB and effect of non-steroidal anti-inflammatory drugs. Cell Cycle.

[18] Chu TH (2014). Celecoxib suppresses hepatoma stemness and progression by up-regulating PTEN. Oncotarget.

[19] Smith WL, Garavito RM, DeWitt DL (1996). Prostaglandin endoperoxide H synthases (cyclooxygenases)-1 and -2. Journal of Biological Chemistry.

[20] Harikumar KB, Aggarwal BB (2008). Resveratrol: a multitargeted agent for age-associated chronic diseases. Cell Cycle.

[21] Orhan H, Dogruer DS, Cakir B, Sahin G, Sahin MF (1999). The *in vitro* effects of new non-steroidal antiinflammatory compounds on antioxidant system of human erythrocytes. Exp Toxicol Pathol.

[22] Vanaja K, Wahl MA, Bukarica L, Heinle H (2013). Liposomes as carriers of the lipid soluble antioxidant resveratrol: evaluation of amelioration of oxidative stress by additional antioxidant vitamin. Life Sci.

[23] Orhan H, Sahin G (2001). *In vitro* effects of NSAIDS and paracetamol on oxidative stress-related parameters of human erythrocytes. Exp Toxicol Pathol.

[24] Qadri SM, Foller M, Lang F (2009). Inhibition of suicidal erythrocyte death by resveratrol. Life Sci.

[25] Sehirli O, Tozan A, Omurtag GZ, Cetinel S, Contuk G, Gedik N, Sener G (2008). Protective effect of resveratrol against naphthalene-induced oxidative stress in mice. Ecotoxicol Environ Saf.

[26] Kalonia H, Kumar P, Kumar A (2011). Licofelone attenuates quinolinic acid induced Huntington like symptoms: possible behavioral, biochemical and cellular alterations. Prog Neuropsychopharmacol Biol Psychiatry.

[27] Salminen A, Kaarniranta K, KauppinenR67 A (2012). Inflammaging: disturbed interplay between autophagy and inflammasomes. Aging (Albany NY).

[28] Chung JH (2012). Using PDE inhibitors to harness the benefits of calorie restriction: lessons from resveratrol. Aging (Albany NY).

[29] Hachiro T, Yamamoto T, Nakano K, Tanaka K (2013). Phospholipid flippases Lem3p-Dnf1p and Lem3p-Dnf2p are involved in the sorting of the tryptophan permease Tat2p in yeast. Journal of Biological Chemistry.

[30] Casamayor A, Torrance PD, Kobayashi T, Thorner J, Alessi DR (1999). Functional counterparts of mammalian protein kinases PDK1 and SGK in budding yeast. Curr Biol.

[31] Kapahi P, Zid BM, Harpe rT, Koslover D, Sapin V, Benzer S (2004). Regulation of lifespan in *Drosophila* by modulation of genes in the TOR signaling pathway. Current Biology.

[32] Wagner N, Kagermeier B, Loserth S, Krohne G (2006). The *Drosophila melanogaster* LEM-domain protein MAN1. European Journal of Cell Biology.

[33] Abe F, Horikoshi K (2000). Tryptophan permease gene TAT2 confers high-pressure growth in *Saccharomyces cerevisiae*. Molecular and Cellular Biology.

[34] Shishkina LN, Shevchenko OG, Zagorskaya NG, Shishkina LN, Zaikov GE, Goloshchapov AN (2010). Influence on the Oxidation Processes Regulation is the Reason for Biological Activity of the Ecdysteroid-Containing Compounds. Handbook of chemistry, biochemistry and biology: new frontiers.

[35] Timmers S, Auwerx J, Schrauwen P (2012). The journey of resveratrol from yeast to human. Aging (Albany NY).

[36] Ayyadevara S, Bharill P, Dandapat A, Hu C, Khaidakov M, Mitra S, Shmookler Reis RJ, Mehta JL (2012). Aspirin Inhibits Oxidant Stress, Reduces Age-Associated Functional Declines, and Extends Lifespan of *Caenorhabditis elegans*. Antioxidants & redox signaling.

[37] Rascon B, Hubbard BP, Sinclair DA, Amdam GV (2012). The lifespan extension effects of resveratrol are conserved in the honey bee and may be driven by a mechanism related to caloric restriction. Aging (Albany NY).

[38] Moskalev A, Shaposhnikov M (2011). Pharmacological inhibition of NF-κB prolongs lifespan of *Drosophila melanogaster*. Aging (Albany NY).

[39] Danilov A, Shaposhnikov M, Plyusnina E, Kogan V, Fedichev P, Moskalev A (2013). Selective anticancer agents suppress aging in *Drosophila*. Oncotarget.

[40] Tootle TL, Spradling AC (2008). Drosophila Pxt: a cyclooxygenase-like facilitator of follicle maturation. Development.

[41] Calabrese V, Cornelius C, Mancuso C, Pennisi G, Calafato S, Bellia F, Bates TE, Giuffrida Stella AM, Schapira T, Dinkova Kostova AT, Rizzarelli E (2008). Cellular stress response: a novel target for chemoprevention and nutritional neuroprotection in aging, neurodegenerative disorders and longevity. Neurochem Res.

[42] Arking R (2000). Forward and reverse selection for longevity in *Drosophila* is characterized by alteration of antioxidant gene expression and oxidative damage patterns. Experimental Gerontology.

[43] Amrit FR, Boehnisch CM, May RC (2010). Phenotypic covariance of longevity, immunity and stress resistance in the *Caenorhabditis* nematodes. PLoS ONE.

[44] Zhao Y, Sun H, Lu J, Li X, Chen X, Tao D, Huang W, Huang B (2005). Lifespan extension and elevated *hsp* gene expression in *Drosophila* caused by histone deacetylase inhibitors. J Exp Biol.

[45] Moskalev A (2007). Radiation-induced life span alteration of *Drosophila* lines with genotype differences. Biogerontology.

[46] Moskalev AA, Plyusnina EN, Shaposhnikov MV (2011). Radiation hormesis and radioadaptive response in Drosophila melanogaster flies with different genetic backgrounds: the role of cellular stress-resistance mechanisms. Biogerontology.

[47] Moskalev A, Shaposhnikov M, Turysheva E (2009). Life span alteration after irradiation in Drosophila melanogaster strains with mutations of Hsf and Hsps. Biogerontology.

[48] Tatar M, Khazaeli AA, Curtsinger JW (1997). Chaperoning extended life. Nature.

[49] Yang P, He XQ, Peng L, Li AP, Wang XR, Zhou JW, Liu QZ (2007). The role of oxidative stress in hormesis induced by sodium arsenite in human embryo lung fibroblast (HELF) cellular proliferation model. J Toxicol Environ Health A.

[50] Cho Y, Park MJ, Park M, Min SS, Yee J, Kim C, Han MS, Han SH (2009). Effects of CAY10404 on the PKB/Akt and MAPK pathway and apoptosis in non-small cell lung cancer cells. Respirology.

[51] Demidenko ZN, Blagosklonny MV (2009). At concentrations that inhibit mTOR, resveratrol suppresses cellular senescence. Cell Cycle.

[52] Blagosklonny MV (2013). Damage-induced aging and perpetual motion. Cell Cycle.

[53] Gems D, Partridge L (2013). Genetics of longevity in model organisms: debates and paradigm shifts. Annu Rev Physiol.

[54] Blagosklonny MV (2008). Aging: ROS or TOR. Cell Cycle.

[55] Koeberle A, Siemoneit U, Buhring U, Northoff H, Laufer S, Albrecht W, Werz O (2008). Licofelone suppresses prostaglandin E2 formation by interference with the inducible microsomal prostaglandin E2 synthase-1. J Pharmacol Exp Ther.

[56] Gupta A, Kumar A, Kulkarni SK (2011). Targeting oxidative stress, mitochondrial dysfunction and neuroinflammatory signaling by selective cyclooxygenase (COX)-2 inhibitors mitigates MPTP-induced neurotoxicity in mice. Prog Neuropsychopharmacol Biol Psychiatry.

[57] Carrizzo A, Forte M, Damato A, Trimarco V, Salzano F, Bartolo M, Maciag A, Puca AA, Vecchione C (2013). Antioxidant effects of resveratrol in cardiovascular, cerebral and metabolic diseases. Food Chem Toxicol.

[58] Zheng LF, Wei QY, Cai YJ, Fang JG, Zhou B, Yang L, Liu ZL (2006). DNA damage induced by resveratrol and its synthetic analogues in the presence of Cu (II) ions: mechanism and structure-activity relationship. Free Radic Biol Med.

[59] Aad G (2011). Search for dilepton resonances in pp collisions at radicals=7 TeV with the ATLAS detector. Phys Rev Lett.

[60] Sengottuvelan M, Deeptha K, NaliniR69 N (2009). Resveratrol ameliorates DNA damage, prooxidant and antioxidant imbalance in 1,2-dimethylhydrazine induced rat colon carcinogenesis. Chem Biol Interact.

[61] Costa RM, Magalhaes AS, Pereira JA, Andrade PB, Valentao P, Carvalho M, Silva BM (2009). Evaluation of free radical-scavenging and antihemolytic activities of quince (*Cydonia oblonga*) leaf: a comparative study with green tea (*Camellia sinensis*). Food and Chemical Toxicology.

[62] Wang C, Qin X, Huang B, He F, Zeng C (2010). Hemolysis of human erythrocytes induced by melamine-cyanurate complex. Biochemical and Biophysical Research Communications.

[63] Takebayashi J, Chen J, Tai A (2010). A method for evaluation of antioxidant activity based on inhibition of free radical-induced erythrocyte hemolysis. Methods in Molecular Biology.

[64] Asakawa T, Matsushita S (1980). Coloring conditions of thiobarbituric acid test for detecting lipid hydroperoxides. Lipids.

[65] Freitas DR, Rosa RM, Moraes J, Campos E, Logullo C, Da Silva Vaz I, Masuda A (2007). Relationship between glutathione S-transferase, catalase, oxygen consumption, lipid peroxidation and oxidative stress in eggs and larvae of Boophilus microplus (Acarina: Ixodidae). Comp Biochem Physiol A Mol Integr Physiol.

[66] Lavara-Culebras E, Munoz-Soriano V, Gomez-Pastor R, Matallana E, Paricio N (2010). Effects of pharmacological agents on the lifespan phenotype of *Drosophila* DJ-1beta mutants. Gene.

[67] Kaplan EL, Meier P, Kotz S, Johnson N (1992). Nonparametric Estimation from Incomplete Observations. Breakthroughs in Statistics.

[68] Fleming TR, O'Fallon JR, O'Brien PC, Harrington DP (1980). Modified Kolmogorov-Smirnov test procedures with application to arbitrarily right-censored data. Biometrics.

[69] Breslow N (1970). A generalized Kruskal-Wallis test for comparing K samples subject to unequal patterns of censorship. Biometrika.

[70] Mantel N (1966). Evaluation of survival data and two new rank order statistics arising in its consideration. Cancer Chemother Rep.

[71] Wang C, Li Q, Redden DT, Weindruch R, Allison DB (2004). Statistical methods for testing effects on “maximum lifespan”. Mechanism of Ageing and Development.

[72] RCoreTeam (2014). R: A Language and Environment for Statistical Computing.

[73] Fisher RA (1922). On the interpretation of χ2 from contingency tables, and the calculation of P. Journal of the Royal Statistical Society.

[74] Yang J-S, Nam H-J, Seo M, Han SK, Choi Y, Nam HG, Lee S-J, Kim S (2011). OASIS: Online Application for the Survival Analysis of Lifespan Assays Performed in Aging Research. PLoS ONE.

[75] Bonilla-Ramirez L, Jimenez-Del-Rio M, Velez-Pardo C (2013). Low doses of paraquat and polyphenols prolong life span and locomotor activity in knock-down parkin Drosophila melanogaster exposed to oxidative stress stimuli: implication in autosomal recessive juvenile Parkinsonism. Gene.

[76] Mann HB, Whitney DR (1947). On a Test of Whether one of Two Random Variables is Stochastically Larger than the Other. The Annals of Mathematical Statistics.

