# Supplementary material for: Influence of non-steroidal anti-inflammatory drugs on Drosophila melanogaster longevity
Source: Oncotarget. 2015 Aug 7;6(23):19428–44. doi: 10.18632/oncotarget.5118 (PMC4637296; doi:10.18632/oncotarget.5118)
Supplement: Supplementary file 1 [file oncotarget-06-19428-s001.pdf]

Influence of non-steroidal anti-inflammatory drugs on *Drosophila melanogaster* longevity

Supplementary Material

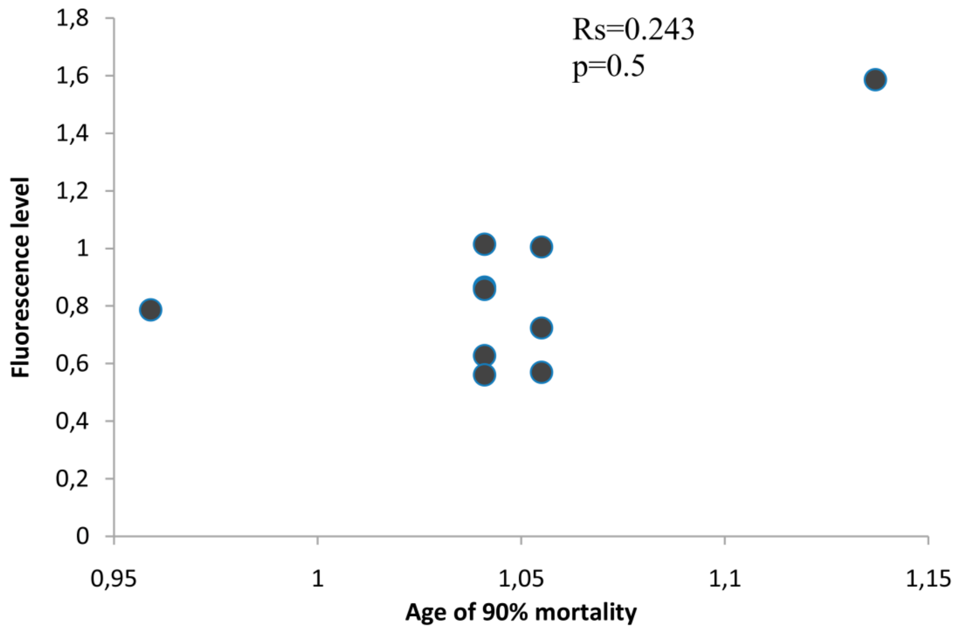

Figure S1.

The relation between the amounts of food consumed and age of 90% mortality of *Drosophila melanogaster* females.

Axis OX - Age of 90% mortality in comparison with control group (control value taken as 1); axis OY - changes of fluorescence level in comparison with control group (control value taken as 1); Rs - Spearman's correlation coefficient; level of significance according to Mann-Whitney test.
